# Association and functional study between *ADIPOQ* and metabolic syndrome in elderly Chinese Han population

**DOI:** 10.18632/aging.104203

**Published:** 2020-11-20

**Authors:** Qiao Wang, Decheng Ren, Yan Bi, Ruixue Yuan, Dong Li, Jianying Wang, Ruirui Wang, Lei Zhang, Guang He, Baocheng Liu

**Affiliations:** 1 Shanghai Innovation Center of Traditional Chinese Medicine Health Service, Shanghai University of Traditional Chinese Medicine, Shanghai, China; 2 Bio-X Institutes of Shanghai Jiao Tong University, Shanghai, China; 3 Zhangjiang Community Health Service Center of Pudong New, Shanghai, China

**Keywords:** metabolic syndrome, ADIPOQ, 3' UTR, association

## Abstract

Objective: Metabolic syndrome (MetS) is a cluster of health problems that places individuals at higher risk of developing cardiovascular disease, diabetes and stroke. The prevalence of MetS is increasing worldwide. It is also well accepted that genetic and environmental factors play significant roles in the occurrence/development of MetS, but studies exploring genetic factors are still lacking. Here, we aimed to investigate the association of *ADIPOQ* gene variants with MetS in an elderly Chinese Han population.

Results: We found that the allelic frequencies of rs6773957 and rs3774261 were significantly different between MetS and the control (*p* = 0.031; *p* = 0.049). Furthermore, a reduction in luciferase activity was observed when HEK293T cells were transfected with rs6773957 mutant fragments compared with wild type.

Conclusion: Our results suggest that rs6773957 and rs3774261 of *ADIPOQ* were associated with MetS in the elderly Chinese Han population. The functional assays performed indicate that the rs6773957 variant might be pathogenic and may provide evidence for mechanistic studies of MetS in the future.

Methods: Four single nucleotide polymorphisms (SNPs) were selected and genotyped (rs6773957, rs182052, rs3774261 and rs17366568) in 1337 subjects, including 569 healthy controls and 768 MetS cases. The clinical characteristics of all the subjects were obtained and analyzed. Additionally, a functional study of rs6773957 in regulating the expression of *ADIPOQ* was performed in this study.

## INTRODUCTION

Metabolic syndrome (MetS), also known as syndrome X or insulin resistance syndrome (IRS), refers to a constellation of metabolic disorders that increase the risk of an individual developing obesity, type 2 diabetes mellitus (T2DM) and atherosclerotic-related disease [1]. The prevalence of MetS was 64% to 73% in subjects with T2DM in Iran [2]. In Taiwan, Hwang et al. found that the prevalence of MetS was positively associated with the degree of obesity [3, 4]. According to a recent meta-analysis, the prevalence of MetS was positively associated with aging [5]. The prevalence of MetS was nearly 35% in adults and 50% in subjects aged ≥ 60 years [6].

The incidence of MetS is often associated with the incidence of T2DM or obesity (outcomes of MetS). In addition, a number of other features have been recently associated with MetS onset, such as nonalcoholic fatty liver disease (NAFLD), polycystic ovarian syndrome, the proinflammatory state, and oxidative stress [7].

The development of this MetS is influenced by a series of environmental and genetic factors. Genetic factors are believed to play an important role in the occurrence of MetS. Eshaghi et al. found that AKT1 polymorphism was associated with major components of MetS [8]. Additionally, the association of genetic variants (*AKT1* rs2494746, *AKT2* rs4802071 and *FRAP1* rs4845856) with risk factors for MetS, such as diabetes and obesity, has been previously reported [9]. Moreover, a recent study reported that the interaction of the *FTO* rs9939609 variant and BMI can significantly increase the risk for MetS among whites over time [10]. However, despite a large number of studies demonstrating strong associations between various SNPs in key genes and MetS, the pathogenesis of this complex metabolic disorder is still unclear.

*ADIPOQ* (adiponectin, C1Q and collagen domain containing), expressed exclusively in adipose tissue, encodes several proteins, such as adiponectin, collagens X and VIII, and complement factor C1q. Numerous SNPs have been found to be associated with MetS, such as *ADIPOQ* rs1501299, rs822396, and rs1501299 polymorphisms. In 2018, Harjit Kaur et al. reported that the *ADIPOQ* rs822396 and rs1501299 polymorphisms and their haplotype combinations were significantly associated with obesity risk and metabolic syndrome parameters in the North Indian Punjabi population [11]. Additionally, the reductions in continuous MetS score were found to be significantly associated with rs1501299 (G/T) in *ADIPOQ* [12]. Although the pathogenesis of MetS is still unclear, researchers have found that insulin resistance (IR) may play a core role in the progression of MetS [13]. In addition, the plasma adiponectin concentration was negatively associated with IR, which indicates that *ADIPOQ* might influence either the incidence or development of MetS [14–16].

In view of the involvement of *ADIPOQ* in the molecular mechanism of MetS and the lack of studies focusing on the association between *ADIPOQ* variants and MetS in the Chinese Han population, we performed an association study to investigate whether the four *ADIPOQ* SNPs (rs182052, rs3774261, rs6773957 and rs17366568) are associated with MetS in the elderly Chinese Han population. Furthermore, we also conducted an *in vitro* functional assay to evaluate the role of *ADIPOQ* rs6773957 in the development of MetS.

## RESULTS

As shown in Table 1, a total of 569 control and 768 MetS subjects were recruited in our study. Individuals with MetS had higher age, BMI, WC, SBP, DBP, FBG, ALT and TG and lower HDL-C compared to levels in those without MetS (*p* < 0.001). AST, TC and LDL-C did not show a significant difference between MetS and the control. The genotypic distribution of all four SNPs obeyed HWE, and the allelic and genotypic distributions are shown in Table 2. Rs3774261 and rs6773957 showed significant differences between MetS and the control in allelic frequencies (*p* = 0.049 vs. *p* = 0.031). The frequency of rs6773957 G was 4.2% higher in the cases than in the controls.

**Table 1:** Clinical and laboratory biochemical characteristics of the study population.

	**Control (n=569)**	**MetS (n=768)**	***p* value**
Male, n (%)	301 (52.9%)	294 (38.3%)	<0.001^*^
Age (years)	71.60±5.31	73.50±5.88	<0.001^*^
BMI (kg/m^2^)	23.33±3.39	26.43±3.17	<0.001^*^
WC (cm)	79.88±8.60	88.63±7.88	<0.001^*^
SBP (mmHg)	133.69±19.83	147.68±19.85	<0.001^*^
DBP (mmHg)	73.78±8.05	77.12±8.93	<0.001^*^
FBG (mmol/L)	5.42±1.08	6.83±2.07	<0.001^*^
ALT (U/L)	19.20±16.55	22.63±15.01	<0.001^*^
AST (U/L)	22.68±16.51	22.41±10.44	0.715
TC (mmol/L)	4.73±0.88	4.81±1.13	0.152
TG (mmol/L)	1.11±0.41	2.11±1.78	<0.001^*^
HDL-C (mmol/L)	1.35±0.27	1.11±0.22	<0.001^*^
LDL-C (mmol/L)	3.00±0.76	3.02±0.83	0.584

Adjusted for male sex, age or BMI in ANCOVA. WC: waist circumference; SBP: systolic blood pressure; DBP: diastolic blood pressure; FBG: fasting blood glucose; ALT: alanine aminotransferase; AST: aspartate transaminase; TC: total cholesterol; TG: triglyceride; HDL-C: high-density lipoprotein cholesterol; LDL-C: low-density lipoprotein cholesterol.

**Table 2:** Genetic association of four SNPs of *ADIPOQ.*

**Gene**	**SNP ID**	**Allele frequency**		**χ^2^**	***p* value**	**Genotype frequency**			**χ^2^**	***p* value**	**HWE *p* value**
*ADIPOQ*	rs182052	A	G	0.003	0.958	A/A	A/G	G/G	0.923	0.630	
	Case	667(0.443)	839(0.557)			147(0.195)	373(0.495)	233(0.309)			0.917
	Control	494(0.442)	624(0.558)			116(0.208)	262(0.469)	181(0.324)			0.239
	rs3774261	A	G	3.879	0.049^*^	A/A	A/G	G/G	3.839	0.147	
	Case	827(0.545)	691(0.455)			227(0.299)	373(0.491)	159(0.209)			0.800
	Control	651(0.583)	465(0.417)			191(0.342)	269(0.482)	98(0.176)			0.845
	rs6773957	A	G	0.842	0.031^*^	A/A	A/G	G/G	4.599	0.100	
	Case	823(0.542)	695(0.458)			226(0.298)	371(0.489)	162(0.213)			0.671
	Control	658(0.584)	468(0.416)			195(0.346)	268(0.476)	100(0.178)			0.634
	rs17366568	A	G	0.549	0.459	A/A	A/G	G/G	0.750	0.687	
	Case	51(0.034)	1469(0.966)			1(0.001)	49(0.064)	710(0.934)			0.872
	Control	32(0.028)	1092(0.972)			1(0.002)	30(0.053)	531(0.945)			0.406

Pearson’s *p* value and significant *p* (<0.05) values are marked.

HWE: Hardy-Weinberg equilibrium.

**Figure 1:**
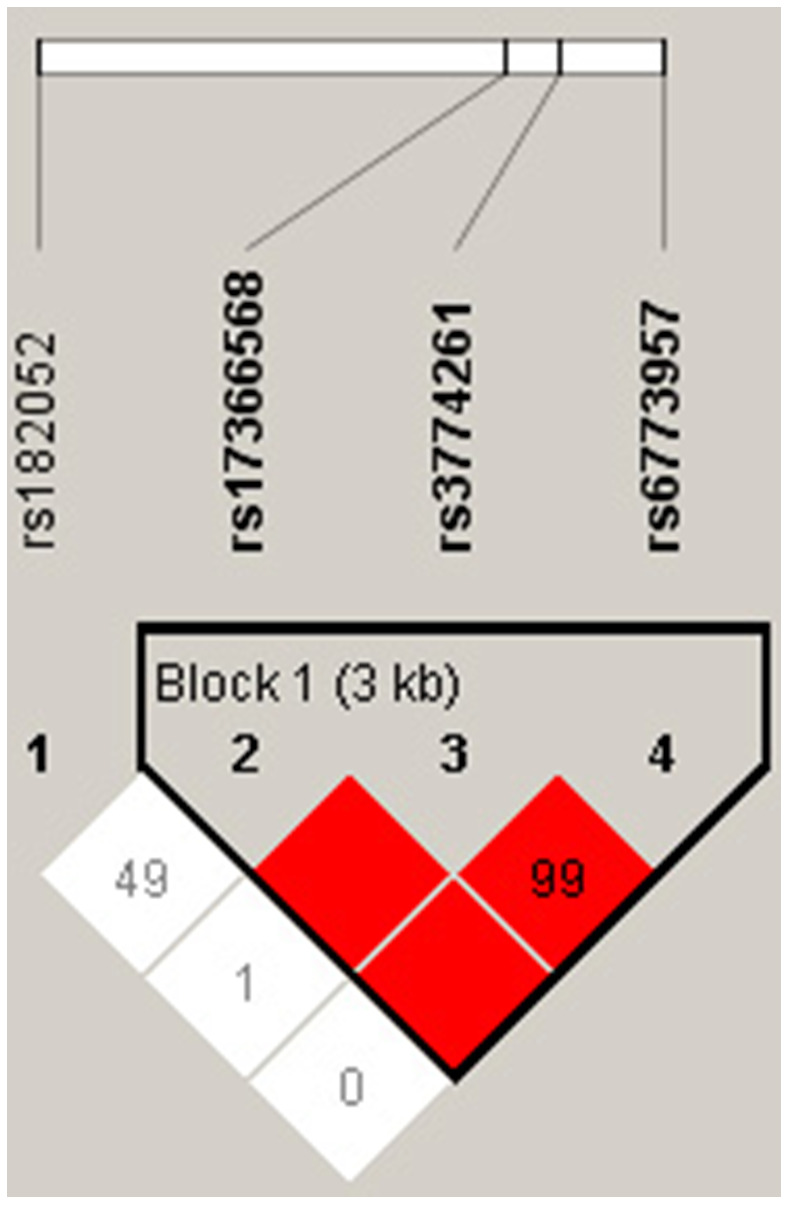
**LD structure of the four polymorphisms in the *ADIPOQ* gene with Haploview analysis.** Red squares indicate significant statistical LD between the pair of polymorphisms (D’ > 80).

Pairwise LD estimates defined by D’ showed strong LD between rs3774261, rs6773957 and rs17366568 (Figure 1). Haplotypes with frequencies less than 3% were excluded from further analysis. The global frequencies of haplotypes with different combinations of four polymorphisms (rs182052-rs3774261-rs6773957-rs17366568) exhibited differences between the case and control groups (*p* = 0.016). The individual haplotypes GAAG^*^ (*p* = 0.020) and GGGG^*^ (*p* = 0.004) showed significant differences between the MetS and control groups (Table 3).

**Table 3:** Haplotype analysis within the block.

**Haplotype**	**Case frequency**	**Control frequency**	**χ^2^**	***p* value**	**Global *p***
rs182052-rs3774261-rs6773957-rs17366568					0.016
G A A G^*^	424.10(0.289)	361.82(0.333)	5.372	0.020	
G G G G^*^	359.08(0.244)	215.10(0.198)	8.264	0.004	

**Figure 2:**
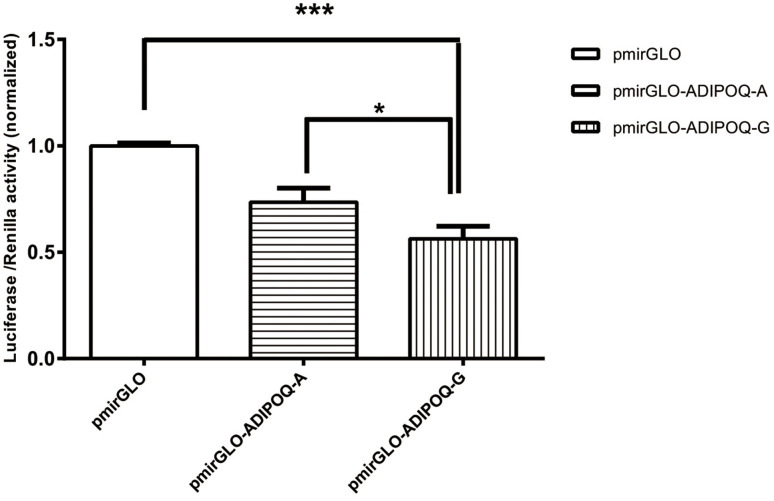
**The luciferase activity of the two recombinant DNA plasmids *ADIPOQ*-A (wild type) and *ADIPOQ*-G (mutant type) transfected in HEK293 cells was graphed after normalization against the plasmid pmirGLO expressing the Renilla luciferase gene.** Data for rs6773957 pmirGLO, pmirGLO-*ADIPOQ*-A and pmirGLO-*ADIPOQ*-G are presented as the mean and SD of n=9 samples. ***, *p* 0.001, one-way ANOVA test.

As shown in Figure 2, a dual-luciferase reporter assay was carried out in HEK293 cells using pmirGlO, pmirGLO-*ADIPOQ*-A, and pmirGLO-*ADIPOQ*-G. There were significant differences between the expression levels of the three vectors (*p* < 0.05). The luciferase expression level of pmirGLO-*ADIPOQ*-A was 17.2% higher than that of pmirGLO-*ADIPOQ*-G, which showed a significant downregulation of luciferase activity compared to pmirGLO-*ADIPOQ*-A (*p* = 0.031).

## DISCUSSION

To investigate the association between *ADIPOQ* SNPs and MetS in the elderly Chinese Han population, a case-control study with 1337 subjects (768 MetS vs. 569 controls) was carried out. Rs3774261 and rs6773957 polymorphisms in the *ADIPOQ* gene were associated with MetS. Additionally, the frequencies of allele G in cases were 4% higher than in controls for both SNPs. According to LD analysis, rs3774261, rs6773957 and rs17366568 were in a strong LD block. This was the first time that rs3774261, rs6773957 and rs17366568 have been reported to be in a haplotype block and additionally found to be associated with MetS. Haplotype GAAG^*^ was a genetic protective factor for MetS (*p* = 0.020), and haplotype GGGG^*^ was positively related to individuals with MetS (*p* = 0.004). In terms of functional analysis, the luciferase expression level of pmirGLO-*ADIPOQ*-G showed a significant downregulation of luciferase activity compared with pmirGLO-*ADIPOQ*-A.

Adiponectin, encoded by the *ADIPOQ* gene, is of particular interest in metabolic syndrome because of its inverse relation with insulin sensitivity and obesity [17]. An increasing number of studies have focused on the association of the *ADIPOQ* polymorphism with IR and MetS. A previous study showed that adiponectin levels influenced insulin sensitivity, which indicated that there might be a causal relationship between adiponectin levels and insulin sensitivity [18]. In addition, experiments conducted in animals found that adiponectin played a vital role in the regulation of insulin sensitivity and that adiponectin was negatively associated with IR [19, 20]. Specchia et al. also found that the *ADIPOQ* gene might have an effect on the phenotype of metabolic traits (lipids, glucose, insulin, and insulin sensitivity) [15]. In our study, we found that the level of triglycerides was higher in patients with MetS than in controls. According to our Dual Luciferase Reporter Gene Assay, the rs6773957 G allele was associated with lower expression of *ADIPOQ*. Furthermore, in our study, we found that patients with MetS had 4.2% more G alleles than controls.

Rs6773957, in the 3’ UTR of *ADIPOQ*, is located on Chr 3: 186855916. Recently, another study found that rs6773957 was associated with plasma adiponectin levels. A whole genome association study reported that rs6773957 and rs3774261 were strongly associated with plasma adiponectin levels [21]. Moreover, new evidence showed that rs6773957 was not only strongly associated with the serum adiponectin concentration but was also positively associated with body weight, which indicated that rs6773957 might have an effect on mRNA stability or translational efficiency [22]. Although several studies have shown an association between *ADIPOQ* and IR, few have shown an association between *ADIPOQ* rs6773957 and MetS. In one such study, the authors found that rs6773957 showed the strongest signal in the 3’UTR of *ADIPOQ* [23]. Additionally, Wang et al. found that carriers of the G allele of rs6773957 had a higher risk of developing MetS [24], which was in line with our result. However, no functional analysis of *ADIPOQ* rs6773957 and MetS was observed in previous studies. Therefore, a dual-luciferase reporter assay was performed to verify whether the rs6773957 polymorphism would influence the expression of the *ADIPOQ* gene. We found that there was a significant difference in luciferase activity that was dependent on the different alleles of rs6773957, which indicates that the expression of *ADIPOQ* was inhibited.

Our results are consistent with some but not all previous association-based studies. Apart from rs6773957, rs182052 was reported to be associated with obesity in Hispanics [25]. In addition, Li et al. found that the rs3774261 polymorphism was associated with genetic susceptibility to NAFLD, which is regarded as the hepatic manifestation of metabolic syndrome [26]. Additionally, Peters found that there was no association observed between rs3774261, rs17366568 and MetS in a Western Australian cohort study [27]. Therefore, due to the number of conflicting studies, further genetic studies with larger sample sizes and more comprehensive SNPs are needed to verify these results. In addition, the function of the protein will be examined in further studies.

In summary, we have demonstrated that *ADIPOQ* is associated with MetS in our study population. We found that rs6773957 in the 3’UTR of *ADIPOQ* is strongly associated with MetS and that the polymorphism might influence the expression of *ADIPOQ*. Future studies are required to characterize the mechanisms by which miRNAs control the transcription of *ADIPOQ* and to validate the hypothesis *in vivo*. Overall, our study provides new insights into the association analysis between genes and MetS and the molecular mechanism underlying MetS, which will provide additional evidence for the treatment of MetS in the future.

## MATERIALS AND METHODS

### Study subjects

In this study, 768 unrelated adult subjects with MetS (294 males and 474 females, age: 73.5±5.9 years) and 569 controls (301 males and 268 females, age: 71.6±5.3 years) were recruited from the Zhangjiang community of Shanghai, China. The subjects were diagnosed with MetS by following a joint scientific statement [28]. Subjects with at least three of the following features were classified as positive for MetS: ① Abdominal obesity: waist circumstance (WC) ≥ 85 cm for females or ≥ 90 cm for males in China; ② Arterial tension: systolic blood pressure (SBP) ≥ 130 mmHg or diastolic blood pressure (DBP) ≥ 85 mmHg, or treatment for hypertension; ③ elevated fasting blood glucose (FBG): ≥ 5.6 mmol/L or diabetes treatment; ④ elevated triglyceride (TG): ≥ 1.7 mmol/L; and ⑤ reduced high-density lipoprotein cholesterol (HDL-C): < 1.0 mmol/L for males or < 1.3 mmol/L for females. The exclusion criteria were as follows: subjects aged < 60 years old, nonpermanent residents, any psychiatric problem, malignant tumors, severe heart diseases, and those with missing anthropometric or metabolic data or incompletely recorded information. Signed informed consent was obtained from each subject. The study protocol was approved by the ethics committee of Shanghai Innovation Center of Traditional Chinese Medicine Health Service and the Shanghai University of Traditional Chinese Medicine. The study was conducted in accordance with the guidelines in the Declaration of Helsinki.

### Genotyping

Four SNPs (rs182052, rs3774261, rs6773957, and rs17366568) were genotyped in our study. Three of them (rs182052, rs3774261, rs17366568) are located in the intron region, whereas SNP rs6773957 is located in the 3’ UTR. These four SNPs were selected to cover the region of the *ADIPOQ* gene, including tag SNPs, functional domains and others (Table 4). Genomic DNA was extracted from peripheral blood samples by using the standard phenol-chloroform method. SNPs were genotyped by matrix-assisted laser desorption/ionization (MALDI) time-of-flight (TOF) mass spectrometry (MS) using a MassARRAY® Analyzer 4 platform (Sequenom, San Diego, CA). The primers and probes for PCR were designed using the online Assay Design Suite v2.0 Sequenom software. We performed standard PCR with 10 ng of genomic DNA in a total volume of 5 μL using Taqman® Universal PCR Master Mix reagent (Applied Biosystems).

**Table 4:** Basic information of the four SNPs of *ADIPOQ*.

**SNP**	**Call rate**	**Position**	**Function**
rs182052	98.13%	3:186842993	intron
rs3774261	98.50%	3:186853770	intron
rs6773957	98.88%	3:186855916	3' UTR
rs17366568	98.88%	3:186852664	intron

### Anthropometric and biochemical measurements

In this study, anthropometric and biochemical measurements were recorded for all subjects in a standardized manner. The weight of the subjects was measured to the nearest 0.1 kg with subjects dressed in light clothing. Height and WC were measured to the nearest 0.1 cm using a standard tape. WC was measured using a standard tape at the midpoint between the lowest rib and the superior border of the iliac crest in the standing position. Body mass index (BMI) was calculated as weight (kg) divided by the square of height (m). SBP and DBP were measured in a seated position using an electronic sphygmomanometer (Biospace; Cheonan, South Korea). Biochemical parameters, such as FBG, total cholesterol (TC), TG, HDL-C, and low-density lipoprotein cholesterol (LDL-C), were measured by an automatic biochemistry analyzer (Hitachi; Tokyo, Japan).

### Reporter plasmid construction

The 3’UTR containing rs6773957 of *ADIPOQ* was synthetized *in vitro*. The variant was produced by site-directed mutagenesis using a Hieff MutTM Site-Directed Mutagenesis Kit (YEASEN, China). The constructed plasmids were confirmed by sequencing. During the synthesis of the target segment, XhoI and XbaI restriction sites were introduced at the 5’ and 3’ ends, respectively. The sequence was purified using the AxyPrep DNA Gel Extraction Kit (Axygen, China) and cloned into the pmirGLO vector (Promega, USA). This vector had two luciferases – Renilla luciferase and firefly luciferase. The former was used as a control reporter for selection and normalization, and the latter acted as the main reporter to monitor mRNA regulation. The cloned sequence is available on request.

### Cell culture and transfection

HEK293 cells were cultured in Dulbecco’s modified Eagle’s medium (DMEM) with 10% fetal bovine serum (FBS), penicillin (100 U/ml) and streptomycin (100 μg/ml) at 37° C in the presence of 5% CO_2_ humidified air. HEK293 cells were passaged every two days. Approximately 24 hours before transfection, cells were seeded in 12-well plates. When the cells reached 70% - 80% confluence, they were transfected with the pmirGLO vector containing fragments of DNA insertion sequences using FuGENE® HD Transfection Reagent (Promega, USA) and Opti-MEM reduced serum medium following the manufacturer’s instructions. Vectors were divided into three groups, namely, pmirGLO without transfection, for which the luciferase expression level was considered 100%, and the two other vectors that were transfected, pmirGLO- *ADIPOQ* -A with wild type and pmirGLO- *ADIPOQ* -G with mutant. Next, the cells were lysed using Passive Lysis Buffer (YEASEN, China). Subsequently, a Dual Luciferase Reporter Gene Assay Kit (YEASEN, China) and a Promega Glomax20/20 luminometer (Promega, USA) were used to record the luciferase activity.

### Statistical analysis

Statistical analyses were performed using SPSS v 25.0 (SPSS, Chicago, USA). Demographic and clinical data presented in this study are reported as the mean along with the standard deviation (SD). Allele and genotype frequencies were compared between the two groups with the chi-square test. Hardy-Weinberg equilibrium (HWE) and allelic and genotypic distributions were calculated using SHEsis (http://analysis.bio-x.cn/). Pairwise linkage disequilibrium (LD) was measured by Haploview. Because there was statistical significance regarding sex and age between the MetS and control subjects, analysis of covariance (ANCOVA) was used to analyze anthropometric indices and chemical parameters. Analysis of variance (ANOVA) was used to compare the normalized relative luciferase/Renilla activities in the different plasmids with GraphPad. *P* values ≤ 0.05 (two-sided) were considered statistically significant.

## References

[1] Swarup S, Goyal A, Grigorova Y, Zeltser R. Metabolic Syndrome. In: StatPearls. Treasure Island (FL): StatPearls Publishing; 2020. 29083742

[2] Foroozanfar Z, Najafipour H, Khanjani N, Bahrampour A, Ebrahimi H. The prevalence of metabolic syndrome according to different criteria and its associated factors in type 2 diabetic patients in Kerman, Iran. Iran J Med Sci. 2015; 40:522–25. 26538781 PMC4628143

[3] Hwang LC, Bai CH, Sun CA, Chen CJ. Prevalence of metabolically healthy obesity and its impacts on incidences of hypertension, diabetes and the metabolic syndrome in Taiwan. Asia Pac J Clin Nutr. 2012; 21:227–33. 22507609

[4] Hwang LC, Bai CH, Chen CJ. Prevalence of obesity and metabolic syndrome in Taiwan. J Formos Med Assoc. 2006; 105:626–35. 10.1016/S0929-6646(09)60161-3 16935763

[5] Li R, Li W, Lun Z, Zhang H, Sun Z, Kanu JS, Qiu S, Cheng Y, Liu Y. Prevalence of metabolic syndrome in Mainland China: a meta-analysis of published studies. BMC Public Health. 2016; 16:296. 10.1186/s12889-016-2870-y 27039079 PMC4818385

[6] Aguilar M, Bhuket T, Torres S, Liu B, Wong RJ. Prevalence of the metabolic syndrome in the United States, 2003-2012. JAMA. 2015; 313:1973–74. 10.1001/jama.2015.4260 25988468

[7] Bruce KD, Hanson MA. The developmental origins, mechanisms, and implications of metabolic syndrome. J Nutr. 2010; 140:648–52. 10.3945/jn.109.111179 20107145

[8] Eshaghi FS, Ghazizadeh H, Kazami-Nooreini S, Timar A, Esmaeily H, Mehramiz M, Avan A, Ghayour-Mobarhan M. Association of a genetic variant in AKT1 gene with features of the metabolic syndrome. Genes Dis. 2019; 6:290–95. 10.1016/j.gendis.2019.03.002 32042868 PMC6997569

[9] Yin X, Xu Z, Zhang Z, Li L, Pan Q, Zheng F, Li H. Association of PI3K/AKT/mTOR pathway genetic variants with type 2 diabetes mellitus in Chinese. Diabetes Res Clin Pract. 2017; 128:127–35. 10.1016/j.diabres.2017.04.002 28477532

[10] Hardy DS, Garvin JT, Mersha TB, Racette SB. Ancestry specific associations of FTO gene variant and metabolic syndrome: a longitudinal ARIC study. Medicine (Baltimore). 2020; 99:e18820. 10.1097/MD.0000000000018820 32028392 PMC7015559

[11] Kaur H, Badaruddoza B, Bains V, Kaur A. Genetic association of ADIPOQ gene variants (-3971A>G and +276G>T) with obesity and metabolic syndrome in North Indian Punjabi population. PLoS One. 2018; 13:e0204502. 10.1371/journal.pone.0204502 30265726 PMC6161880

[12] Lowry DE, Fenwick PH, Roke K, Jeejeebhoy K, Dhaliwal R, Brauer P, Royall D, Tremblay A, Klein D, Mutch DM. Variants in APOA5 and ADIPOQ moderate improvements in metabolic syndrome during a one-year lifestyle intervention. Lifestyle Genom. 2018; 11:80–89. 10.1159/000494331 30472712

[13] Li X, Wei D, He H, Zhang J, Wang C, Ma M, Wang B, Yu T, Pan L, Xue F, He H, Xu W, Pan T, et al. Association of the adiponectin gene (ADIPOQ) +45 T > G polymorphism with the metabolic syndrome among Han Chinese in Sichuan province of China. Asia Pac J Clin Nutr. 2012; 21:296–301. 22507618

[14] Yao M, Wu Y, Fang Q, Sun L, Li T, Qiao H. Association of ADIPOQ variants with type 2 diabetes mellitus susceptibility in ethnic Han Chinese from northeast China. J Diabetes Investig. 2016; 7:853–59. 10.1111/jdi.12535 27181706 PMC5089947

[15] Specchia C, Scott K, Fortina P, Devoto M, Falkner B. Association of a polymorphic variant of the adiponectin gene with insulin resistance in African Americans. Clin Transl Sci. 2008; 1:194–99. 10.1111/j.1752-8062.2008.00055.x 20443850 PMC5350656

[16] Wassel CL, Pankow JS, Jacobs DR Jr, Steffes MW, Li N, Schreiner PJ. Variants in the adiponectin gene and serum adiponectin: the coronary artery development in young adults (CARDIA) study. Obesity (Silver Spring). 2010; 18:2333–38. 10.1038/oby.2010.85 20395949 PMC4970734

[17] Henneman P, Aulchenko YS, Frants RR, Zorkoltseva IV, Zillikens MC, Frolich M, Oostra BA, van Dijk KW, van Duijn CM. Genetic architecture of plasma adiponectin overlaps with the genetics of metabolic syndrome-related traits. Diabetes Care. 2010; 33:908–13. 10.2337/dc09-1385 20067957 PMC2845050

[18] Gao H, Fall T, van Dam RM, Flyvbjerg A, Zethelius B, Ingelsson E, Hägg S. Evidence of a causal relationship between adiponectin levels and insulin sensitivity: a mendelian randomization study. Diabetes. 2013; 62:1338–44. 10.2337/db12-0935 23274890 PMC3609596

[19] Kubota N, Terauchi Y, Yamauchi T, Kubota T, Moroi M, Matsui J, Eto K, Yamashita T, Kamon J, Satoh H, Yano W, Froguel P, Nagai R, et al. Disruption of adiponectin causes insulin resistance and neointimal formation. J Biol Chem. 2002; 277:25863–66. 10.1074/jbc.C200251200 12032136

[20] Hotta K, Funahashi T, Bodkin NL, Ortmeyer HK, Arita Y, Hansen BC, Matsuzawa Y. Circulating concentrations of the adipocyte protein adiponectin are decreased in parallel with reduced insulin sensitivity during the progression to type 2 diabetes in rhesus monkeys. Diabetes. 2001; 50:1126–33. 10.2337/diabetes.50.5.1126 11334417

[21] Ling H, Waterworth DM, Stirnadel HA, Pollin TI, Barter PJ, Kesäniemi YA, Mahley RW, McPherson R, Waeber G, Bersot TP, Cohen JC, Grundy SM, Mooser VE, Mitchell BD. Genome-wide linkage and association analyses to identify genes influencing adiponectin levels: the GEMS study. Obesity (Silver Spring). 2009; 17:737–44. 10.1038/oby.2008.625 19165155 PMC4028785

[22] Siitonen N, Pulkkinen L, Lindström J, Kolehmainen M, Eriksson JG, Venojärvi M, Ilanne-Parikka P, Keinänen-Kiukaanniemi S, Tuomilehto J, Uusitupa M. Association of ADIPOQ gene variants with body weight, type 2 diabetes and serum adiponectin concentrations: the Finnish diabetes prevention study. BMC Med Genet. 2011; 12:5. 10.1186/1471-2350-12-5 21219602 PMC3032655

[23] Hivert MF, Manning AK, McAteer JB, Florez JC, Dupuis J, Fox CS, O'Donnell CJ, Cupples LA, Meigs JB. Common variants in the adiponectin gene (ADIPOQ) associated with plasma adiponectin levels, type 2 diabetes, and diabetes-related quantitative traits: the Framingham offspring study. Diabetes. 2008; 57:3353–59. 10.2337/db08-0700 18776141 PMC2584143

[24] Wang R, Sun lj, Yin F, Lu Q, Shen Y, Wu G, Liu B. Association of ADIPOQ rs6773957 (A/G) and Metabolic Syndrome. Chinese Journal of Internal Medicine. 2010; 49:328–9. 10.3760/cma.j.issn.0578-1426.2010.04.016

[25] Sutton BS, Weinert S, Langefeld CD, Williams AH, Campbell JK, Saad MF, Haffner SM, Norris JM, Bowden DW. Genetic analysis of adiponectin and obesity in Hispanic families: the IRAS family study. Hum Genet. 2005; 117:107–18. 10.1007/s00439-005-1260-9 15843989

[26] Li HJ, Li CP, Zhang C, Zhong XL, Shi L. Association of Adiponectin gene polymorphisms and nonalcoholic fatty liver disease. Int J Clin Exp Med. 2015; 8:16676–81. 26629202 PMC4659090

[27] Peters KE, Beilby J, Cadby G, Warrington NM, Bruce DG, Davis WA, Davis TM, Wiltshire S, Knuiman M, McQuillan BM, Palmer LJ, Thompson PL, Hung J. A comprehensive investigation of variants in genes encoding adiponectin (ADIPOQ) and its receptors (ADIPOR1/R2), and their association with serum adiponectin, type 2 diabetes, insulin resistance and the metabolic syndrome. BMC Med Genet. 2013; 14:15. 10.1186/1471-2350-14-15 23351195 PMC3598639

[28] Alberti KG, Eckel RH, Grundy SM, Zimmet PZ, Cleeman JI, Donato KA, Fruchart JC, James WP, Loria CM, Smith SC Jr, and International Diabetes Federation Task Force on Epidemiology and Prevention, and Hational Heart, Lung, and Blood Institute, and American Heart Association, and World Heart Federation, and International Atherosclerosis Society, and International Association for the Study of Obesity. Harmonizing the metabolic syndrome: a joint interim statement of the International Diabetes Federation Task Force on Epidemiology and Prevention; National Heart, Lung, and Blood Institute; American Heart Association; World Heart Federation; International Atherosclerosis Society; and International Association for the Study of Obesity. Circulation. 2009; 120:1640–5. 10.1161/CIRCULATIONAHA.109.192644 19805654

